# Attraction to water and polarization in dragonflies and damselflies along a light pollution gradient

**DOI:** 10.1093/beheco/arag108

**Published:** 2026-08-27

**Authors:** Valentina Sandoval-Granillo, Angélica S Ensaldo-Cárdenas, Bruce A Robertson, Giovanna Villalobos-Jimenez, Alex Córdoba Aguilar

**Affiliations:** Posgrado en Ciencias Biológicas, Universidad Nacional Autónoma de México, Av. Ciudad Universitaria 3000, Coyoacán, Mexico City 04510, Mexico; Instituto de Ecología, Universidad Nacional Autónoma de México, Apdo. Postal 70-275, Circuito Exterior, Ciudad Universitaria, Coyoacán, México City 04510, Mexico; Posgrado en Ciencias Biológicas, Universidad Nacional Autónoma de México, Av. Ciudad Universitaria 3000, Coyoacán, Mexico City 04510, Mexico; Instituto de Ecología, Universidad Nacional Autónoma de México, Apdo. Postal 70-275, Circuito Exterior, Ciudad Universitaria, Coyoacán, México City 04510, Mexico; Biology Program, Bard College, Annandale-on-Hudson, NY 12504, United States; Centro de Investigación en Ciencias de Información Geoespacial, Circuito Tecnopolo Norte, No. 107, Col. Tecnopolo Pocitos II, Aguascalientes, Aguascalientes 20313, Mexico; Instituto de Ecología, Universidad Nacional Autónoma de México, Apdo. Postal 70-275, Circuito Exterior, Ciudad Universitaria, Coyoacán, México City 04510, Mexico

**Keywords:** polarized light pollution, ecological trap, odonata, light pollution gradient, aquatic insects, water perception

## Abstract

Aquatic insects, like odonates, rely on polarized light as a visual cue to locate water bodies for reproduction. However, artificial polarization from light pollution can lead them into ecological traps. This study investigates odonate preference for polarized light cues compared with other sensory cues associated with water perception, and the effects of exposure to artificial polarization on their sensitivity to these cues. This study tests whether artificial polarization imposes a selective pressure that modifies odonate behavior and sensitivity to water and polarization signals. We predicted that odonate species in areas with higher light pollution would show a reduced preference for anthropogenic polarizing traps and an increased preference for natural water bodies. Our multiple-choice experiment revealed interspecific differences in cue attraction, with some species preferring polarizing traps while others favored water or color cues. Species attracted to the ecological light traps were always a subset of the total species present in each locality, indicating that most species are not attracted to ecological traps. Surprisingly, we found few significant differences in odonate behavior between polarization exposure categories, suggesting either weak selective pressure, prioritization of other sensory cues, or existing physiological-behavioral strategies that allowed them to avoid these ecological traps. We observed sex disparities in susceptibility to all experimental surfaces, with males showing greater attraction, potentially due to differences in habitat selection criteria. While evidence of strong selective pressure from light pollution is lacking, a reduction in light pollution near water bodies is needed to conserve odonates and aquatic insect populations.

## Teaser

Not all that glitters is gold. Polarization mistaken for water fools some odonate species, but others can tell water from human-made materials and escape traps. Light pollution in the form of polarization affects many aquatic insects that reproduce in natural water bodies but it doesn’t seem to impose a strong selective pressure on the entire dragon- and damselfly community. Stricter habitat choice criteria and sex may buffer harm from this type of light pollution.

## Introduction

Polarization is a property of light that describes the orientation of its electric field oscillations. Unpolarized light—such as sunlight—has electric fields vibrating in random directions perpendicular to the direction of propagation. Light becomes polarized when these oscillations are restricted to a single direction or plane.

The perception of polarized light is a key sensory cue used by many insects to orient themselves and navigate their environment. In aquatic insects, this cue is particularly important for detecting water bodies, which serve as critical sites for feeding and reproduction (Schwind 1991; Horváth and Kriska 2008). Shallow water surfaces are the primary natural source of visible and UV range polarized light, and characteristics unique to each body of water—such as depth, transparency, substrate color, illumination, and turbidity—affect how light is reflected and polarized (Horváth and Varjú 2004). This is especially important for aquatic insects like odonates (dragonflies, suborder Anisoptera, and damselflies, suborder Zygoptera) which rely primarily on visual cues to detect water bodies, due to their large compound eyes and reduced antennae (Corbet 1999). In addition, odonates have demonstrated sensory capacities that enable them to assess other water characteristics as part of their habitat choice criteria (Frati et al. 2016). However, the relative importance of these water-related cues compared with polarization signals is still unclear.

An ecological trap emerges when anthropogenic change decouples a signal from its fitness benefits, as a result, a species’ signal reception system can lead it to maladaptive behaviors with which it favors the worst option available (Orians and Wittenberger 1991; Schlaepfer et al. 2002). An organism's response to a novel signal can be either attraction, evasion, or neutrality (Greggor et al. 2019) and natural selection favors an attraction to beneficial options, and avoidance in response to cues that precede harm or danger (Schorkopf et al. 2011; Holderied, et al. 2018). Anthropogenic materials like asphalt, glass, car bodies, vehicle windshields, and solar panels have surfaces that act as sources of light polarization in the visible and the UV ranges both under sunlight and artificial nighttime lighting, a phenomenon which, under the perception of the insect eye, can mimic bodies of water (Horváth et al. 2009). This type of light pollution can work as an ecological trap that lures and reduces aquatic insect survival, leading to reproductive failure and even death (Bernáth et al. 2001).

More than 300 species of aquatic insects including mayflies, caddisflies, tabanid flies, chironomids, stoneflies and odonates are known to exhibit positive polarotaxis or attraction to horizontally polarized light when searching for water (Schwind 1985; Wildermuth 1998; Bernáth et al. 2001; Wildermuth and Horváth 2005; Csabai et al. 2006; Horváth et al. 2007, 2014; Kriska et al. 2008; Lerner et al. 2008; Horváth and Kriska 2008). Artificial polarizing surfaces can act as super-stimuli to these insects since natural water bodies polarize 30% to 70% of the light that they reflect, while man-made objects commonly polarize 80% to 99% of this light, so organisms attracted to higher degrees of polarized light show a higher attraction to these artificial sources (Horváth et al. 1998, 2014; Kriska et al. 1998). Car paintworks, for example, differ in their polarizing properties, and red and black cars exhibit high polarization in the green and the blue, rendering them attractive surfaces for aquatic insect oviposition in contrast to cars painted with lighter colors (Kriska et al. 2006). Male dragonflies can perch above such polarizing surfaces, and defend them against conspecific males (Horváth et al. 2007). Furthermore, ultraviolet (UV) light is often polarized along with light in the visible range on these surfaces and some aquatic insects that detect such UV range of polarization are attracted to this light spectrum (Fraleigh et al. 2021). Many of these human-made polarizing surfaces, which are particularly abundant in human settlements, could heat up considerably when exposed to sunlight due to their dark hue or the materials from which they are made. Thus, failure to perceive that a surface presents a dangerously high temperature could pose an additional threat to insects that spend extended periods in their proximity. The response that insects show to accelerated human-driven change in the environment is crucial for understanding the viability of their populations, especially given the many threats that insects currently face (Halsch et al. 2021). Since ecological traps are an extreme form of behavioral maladaptation, they can either provoke population collapse or lead to population persistence by existing phenotypic plasticity or the occurrence of a rapid evolutionary response (Fletcher et al. 2012).

We aimed to test potential differences between the relative attractiveness of polarized light visual cues and other sensory cues associated with the perception of water in odonates, which are obligate aquatic insects. Additionally, and to elucidate possible thermal harm in these insects from excessive heating of the ecological trap, we evaluated the temperature difference between natural and artificial experimental conditions, including those with or without water. Furthermore, we sought to determine whether increased exposure to artificial polarization caused by higher urbanization exerts a selective pressure, altering odonate responses to either polarized light cues or water-related cues. As these insects might have already suffered selection in urban environments in response to ecological traps, we expected odonate species inhabiting sites with a higher exposure to this light pollution to exhibit a reduced preference for anthropogenic polarizing traps and a higher preference for natural water bodies compared with species in conserved sites with minimal to no light pollution, where we expected a higher attraction to ecological traps.

## Methods

### Study site

This study was carried out in 4 localities near the Chamela-Cuixmala Reserve in Jalisco, Mexico (19°29′54″N, 105°02′39″W). The native vegetation is composed mainly of tropical deciduous forests. The climate is categorized as warm tropical subhumid, marked by a lengthy dry season ranging from November to June (IBUNAM, 2019). The average annual rainfall is 748 mm, with a maximum temperature of 37 °C (Flores-Casas and Ortega-Huerta 2019). Human activities have transformed the surroundings of the Chamela-Cuixmala Reserve, resulting in a mosaic composed of old-growth forests, secondary vegetation, agricultural fields, and human settlements with different levels of urbanization (Sánchez-Azofeifa et al. 2009).

### Polarization gradient

Four localities were established in the municipalities of Villa Purificación and La Huerta, according to a habitat classification based on tree cover and presence of urban features (see: Castillo-Pérez et al. 2022). The localities represent a gradient from high tree cover and low urbanization, to low tree cover and higher urbanization: (i) a river and seasonal streams in a conserved site inside the Chamela-Cuixmala Reserve that presented no urbanization; (ii) a lake and a river in close vicinity to a small settlement of 38 houses in La Mesa; (iii) water pools adjacent to the highway that provides access to the town of Pérula; and (iv) an irrigation canal that crosses the town of Francisco Villa. To verify if this urbanization gradient also represented an artificial polarization gradient, we drew a polygon with a radius of 1.3 km around each locality coordinate using QGIS (QGIS Development Team 2023), in order to define areas that accurately captured the complexity of the water body and surroundings of each site. These polygons were then subdivided into a 20 × 20 m grid, a scale that allowed for detailed imagery. The centroids for each of the 20 × 20 m squares were calculated and their coordinates were extracted. These coordinates were used for obtaining high-resolution aerial satellite images using the “RgoogleMaps” package (Loecher and Ropkins 2015) in R by accessing the Google Static Maps API on 13 February 2024. Since cars, asphalt, glass panes, solar panels, black marble, plastic sheets and oil spills are the primary sources of polarized light pollution because of their smooth and dark surfaces (Horváth et al. 2009), we considered cars to be the most abundant source of artificial polarization in low populated towns such as our study localities. Also, parking lots or spaces where cars congregate can instantly produce a large ecological trap since each car's artificial polarization is summed (Kriska et al. 2006). Stemming from this and in order to have an approximate measure of the degree of artificial polarization in the study areas, we used an automated car detector according to Villalobos-Jiménez et al. (in prep). The cars were remotely and automatically located and counted in satellite imagery using a model trained with YOLOv4, which is a CNN-based object detector developed by Bochkovskiy et al. (2020). The model has a precision of 0.83 and a recall of 0.90 (see Villalobos-Jiménez et al. in prep.). This analysis gave us a total number of cars for each of the localities (Table 1), which was used to corroborate that the different locations also constituted a light pollution gradient. Differences between the car counts at the Chamela-Cuixmala reserve (a preserved area dedicated to conservation) and La Mesa town (a small settlement with around 40 houses and streets with partial vegetation removal) were considered to be significant at the insect scale, and since the presence or absence of a variable in biological terms is an important distinction, we decided to keep them as different categories.

**Table 1:** Urbanization and polarization categories of study sites.

Locality	Forest (%)	Number of cars	Artificial polarization exposure
Chamela-Cuixmala reserve	85.7	0	Null
La Meza town	35.2	10	Light
Pérula highway	18.0	137	Moderate
Villa Zapata	5.0	257	High

Percentage of forest cover (taken from Castillo-Pérez et al. 2022), total satellite image car count, and the resulting polarization categories of the 4 study sites.

The experiment consisting of a multiple treatment choice of 4 experimental surfaces differing in their polarizing properties was performed in 5 sites within each locality, next to a lotic or lentic water body and separated 100 m from each replicate to maximize the independence of the data and reduce the probability of counting the same individual several times on different days. Odonate males have shown high site fidelity if they achieve a mating, if they are older or if they occupy a high-quality territory and most of them return within 3 meters of their previous site on consecutive days (Issa 2024). Additionally, zygopterans in general disperse small distances, and in mark-recapture studies, most of them have been recaptured no further than 100 m from the sites where they were marked, even after the traumatic event of handling (Utzeri et al. 1984; Stettmer 1996).

### Polarization treatments

**Figure 1:**
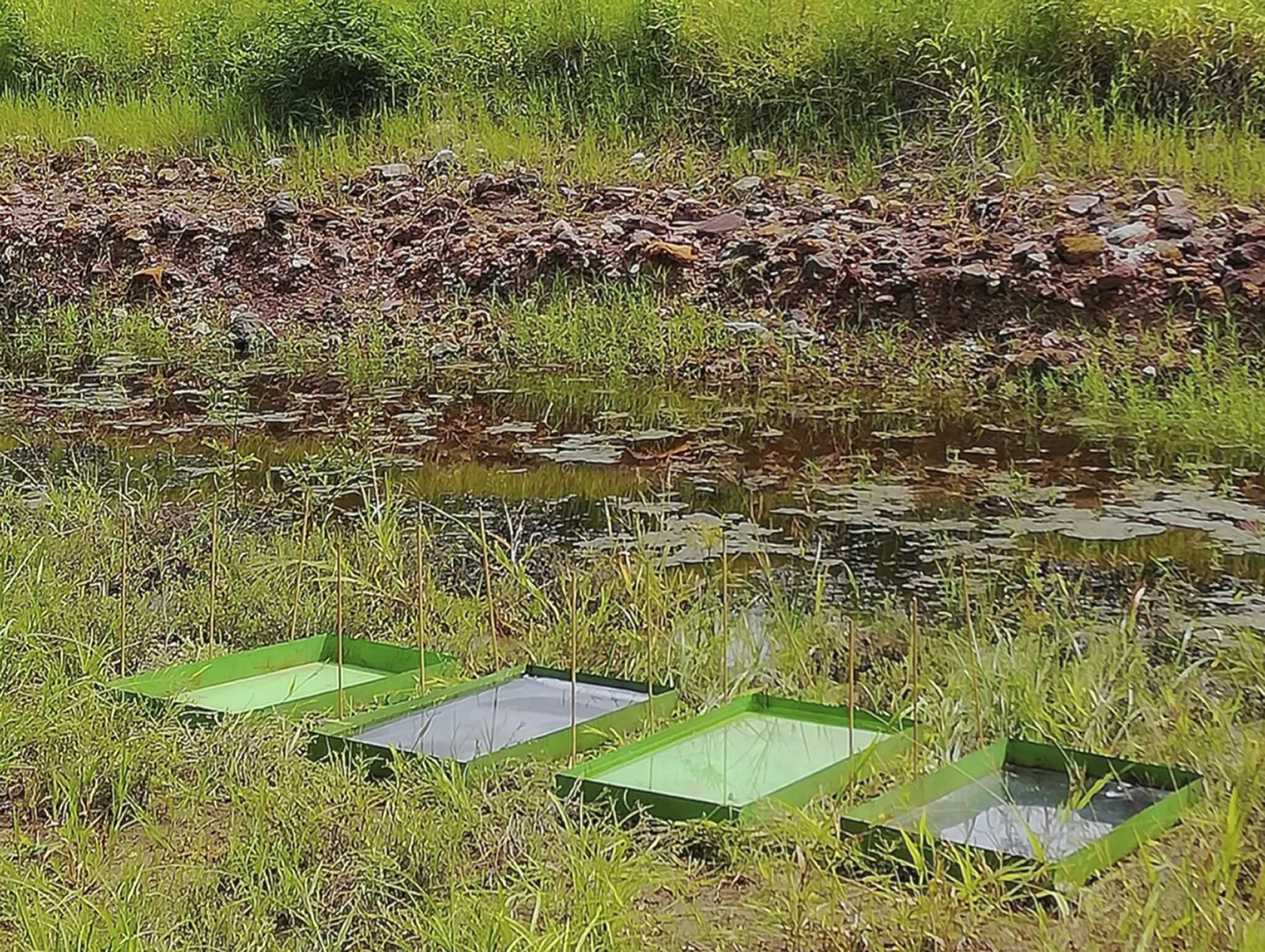
Photograph of the 4 experimental test surfaces next to a natural water body in a site belonging to the moderate polarization category. From left to right: Greenpol, Watermetal, Waterpol and Metal.

**Figure 2:**
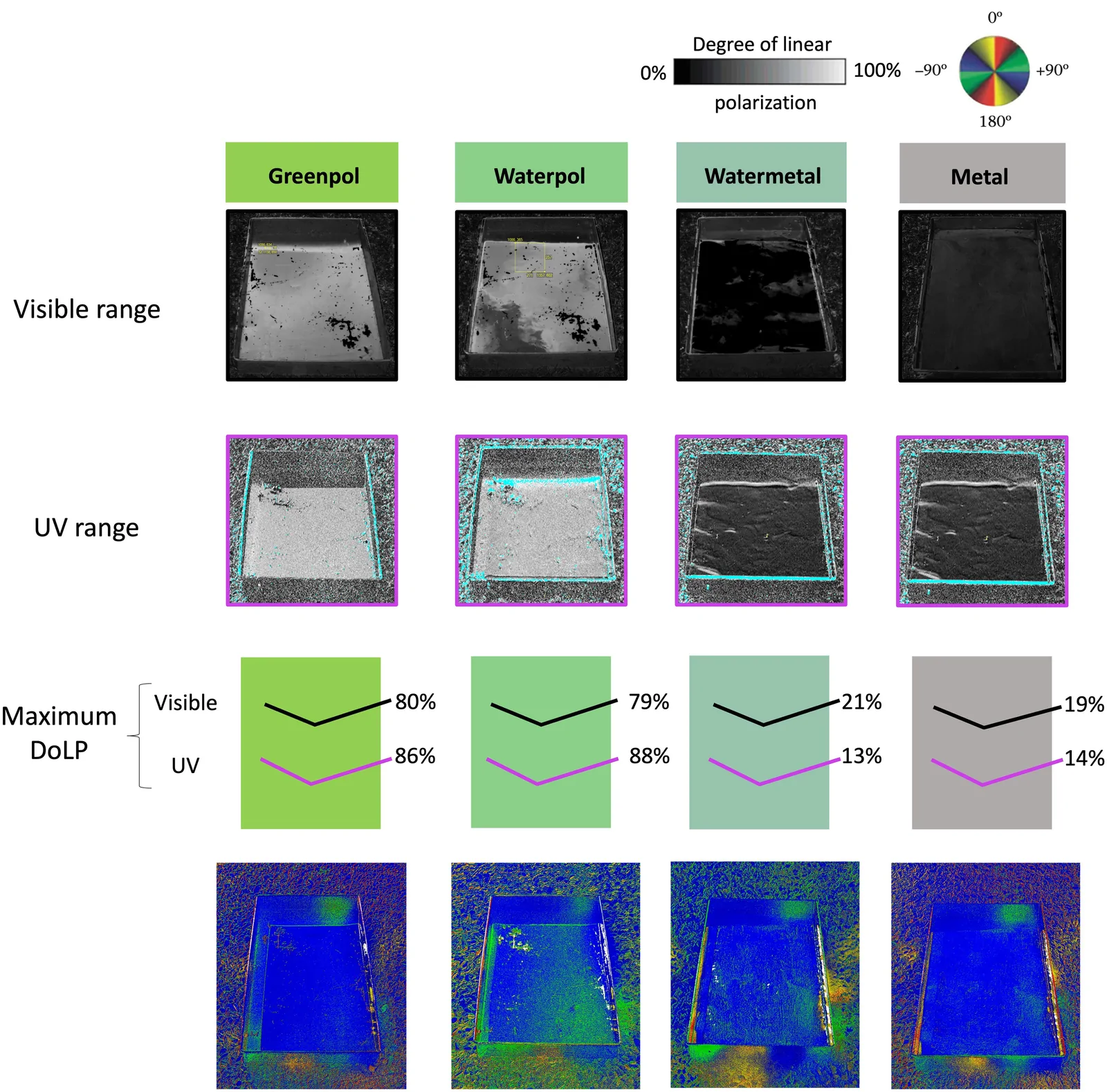
Image polarimetry of the 4 polarization treatment surfaces: (1) metal: tray with a shiny stainless-steel plate covering the base; (2) watermetal: tray with a shiny stainless-steel plate covering the base filled with 8 cm of water; (3) waterpol: tray painted green filled with 8 cm of water; (4) greenpol: empty tray painted green. The first row shows the DoLP of each treatment in the visible range and the second row shows the DoLP in the ultraviolet range, with black being 0% and white being 100% of linear polarization. The maximum degree of polarization in the visual and UV range for each treatment is shown in the third row, with a graphical representation of visible and UV light being reflected off of each surface. The fourth row shows angle of polarization with blue-green being horizontal polarization. The imagery was taken after the experiments were made and chipping of the green paint can be seen as small darker lower polarization spots in the Greenpol and Waterpol images, revealing the metal underneath and evidencing the role of the green paint in increasing polarization.

We designed 4 experimental test surfaces as part of a multiple-choice experiment to evaluate the ability of odonates to differentiate between anthropogenic materials and water with similar polarization properties (Figs 1 and 2). Our goal was to use 2 surfaces with high and low polarization whose ability to polarize light was unaffected by the presence/absence of water, hence creating 2 highly polarizing surfaces, 1 with water and 1 without water, and 2 low polarizing surfaces also with and without water. These surfaces were comprised of rectangular trays of galvanized steel sheets (51 × 90 cm, 8 cm deep, 0.38 mm thick) painted green “pastizal” (Vinimex, Mexico City, Mexico, catalog no. 217-06). The treatments used were: (i) treatment “Greenpol” which consisted of an empty green tray and had a maximum degree of linear polarization (DoLP) of 80% in the visible and 86% in the UV spectrum; (ii) treatment “Waterpol” which included the green tray filled with water to a depth of 8 cm and a maximum DoLP of 79% in the visible and 88% in the UV spectrum: both treatment trays were painted green to look less like man-made objects while maintaining a high polarization due to their darker hue; (iii) treatment “Watermetal” which was the tray with a shiny stainless-steel plate covering the base and was filled with water to a depth of 8 cm, with a maximum DoLP of 21% in the visible and 13% in the UV spectrum; and finally (iv) treatment “Metal” which was the tray with the shiny stainless-steel plate covering the base and no water added: this treatment had a maximum DoLP of 19% in the visible and 14% in the UV spectrum. Tap water was used to fill the Watermetal and Waterpol trays to avoid between-site changes in water properties that could affect the polarized light generated by the trays. The 4 trays were placed 50 cm from the water body in each site, and 25 cm from each other, occupying an area of 2.5 m^2^ and their relative positions were randomized in each site. Three wooden pine sticks, 45 cm high and 0.5 cm thick, were placed vertically along one of the 90 cm edges at 30 cm intervals to serve as perches for the anisopterans and the largest species of zygopterans. The 2.5 m^2^ plots were placed on sites exposed to natural sunlight with as little surrounding vegetation as possible to ensure the uniformity of lighting conditions within and among the experimental plots.

Imaging polarimetry in the visible spectrum was taken using a 4-pol. Thorlabs Kiralux Polarization 4-pol camera 5 MP Monochrome camera (model CS505MUP1, Kamegaki et al. 2024) and 16 to 100 macro zoom lens placed on a tripod. The camera's sensitivity covers human visual range (400 to 700 nm). We imaged the trays aimed sunward under clear skies with the camera aimed downward at the Brewster angle of maximum polarization for water (54°) and the bare aluminum metal and painted trays (52°) which we found to be similar by sampling max polarization of pixels across angles from 48° to59°. Maximum DoLP of light (400 to 700 nm) polarized off the tray bottoms was estimated by subsampling 5 separate circular 500 pixel subsamples of the visual field along the horizontal midline of the Thorcam software (Thorlabs version 3.7.06, https://www.thorlabs.com/software_pages/ViewSoftwarePage.cfm?Code=ThorCam) visualization of pixel polarization to align measurements with the brewster angle. Underexposed sections of the photographs were not considered in calculating the DoLP values. For the polarimetry images in the UV spectrum, we adapted a Nikon DSLR to operate as a UV polarimeter: (i) we replaced a near-pass optical filter with a custom UV-transmitting linearly polarizing filter (Life-Pixel Ltd, https://www.lifepixel.com/) that captured wavelengths above 308 nm and blocked longer, visible wavelengths (>380 nm) and (ii) attached a 60 nm UV-transmitting crystalline macro lens (after Szaz et al. 2016; Fraleigh et al. 2021). We created a custom rotating polarization filter from a Kaesemann HNPB sheet polarizer that polarized transmitted light from 250 to 750 nm. These images were inverted to show white instead of black for 100% polarization so they would coincide with the visible range imagery. Processing of UV imagery was done using AlgoNet software (software developed by Estrato Research & Development Ltd. Used as in Horváth and Varjú 2004) and, following our methods for estimating maximum polarization of tray-reflected light, we subsampled 5, 200-pixel areas along the horizontal mid-line of the processed image using the software's image mask option to locate the area of the highest average DoLP. Algonet software was calibrated to the camera's sensor sensitivity across wavelengths and compensate for these to linearize its intensity scale which could otherwise bias polarization measurements (Foster et al. 2018).

### Behavioral observations and temperature measurements

Two behavioral observations were made per day at each of the 20 sampling sites between 1100 and 1400 h (time of peak activity for adult Odonata). Each observation lasted 50 min, during which we monitored the activity of odonates that interacted with any 1 of the 4 experimental test treatment surfaces. The specific treatment to which each behavior was assigned was established when the individual either stood directly on the tray, perched next to it, or flew directly above it. For each observed individual, the species, sex, treatment, and frequency of the following behaviors were recorded: (i) perching when an insect stood on one of the stick perches or directly on top of the tray, a behavior that allows for monitoring the surroundings; (ii) patrolling, when a male dragonfly hovered over the water body, a behavior related to finding females; and (iii) aggression, when 2 males interacted with agonistic behavior determined by a chase or by confrontation.

Each day at 1300 h a 20 meter transect was established parallel to the water body, with the experimental surface positioned at its midpoint. The observer (VS-G) walked the transect at a consistent pace, completing the 20 meters in 3 min. During each walk, all odonate individuals observed within a 6-meter range on either side of the transect were recorded and counted. At the end of each experiment, other insects that fell in the trays were photographed and collected if they had been killed by the treatment surfaces. Since the vast majority of the subjects were males (130 vs 6 females), the data analyses were made by exclusively considering the male subjects.

### Data analysis

Data were analyzed using R Core Team version 2024.12.1 (R Core Team 2025) using the glmmTMB (Brooks et al. 2017), performance (Lüdecke et al. 2021), emmeans (Lenth 2002) and MuMIn (Bartoń et al. 2025) packages.

### Behavioral observation analysis

To test whether individual species differed in their responses to habitat cues related to water availability and surface polarization, we analyzed the reproductive behaviors of the 4 most abundant odonate species: *Erythrodiplax funerea*, *Orthemis ferruginea*, *Orthemis discolor* (all Anisoptera), and *Telebasis salva (*Zygoptera*)*. For each species we fitted a set of generalized linear mixed models (GLMM) with a negative-binomial error distribution and a log link function implemented with the glmmTMB package (Brooks et al. 2017). The models were:

Model 1 (interaction water and surface): *B* = *β*_0_ + *β*_1_*W* + *β*_2_*S*  *+*  *β*_3_(*W* × *S*)*+ υ*_0_

Model 2 (additive water and surface): *B* = *β*_0_ + *β*_1_*W* + *β*_2_*S*  *+*  *υ*_0_

Model 4 (category only): *B* = *β*_0_ + *β*_1_*S*  *+*  *υ*_0_

Model 3 (surface only): *B* = *β*_0_ + *β*_1_*S*  *+*  *υ*_0_

Model 4 (water only): *B* = *β*_0_ + *β*_1_*W*  *+*  *υ*_0_

Model 5 (null): *B* = *β*_0_ + *υ*_0_

Where *B* represents the total number of reproductive behaviors (ie, perching, patrolling, aggression), *β*_0_ is the model intercept, *W* is the variable denoting water presence or absence, *S* is the variable representing treatment surface (green or steel) and *υ*_0_ is the random intercept of site, included to account for spatial nonindependence among observations. Model diagnostics were assessed using the performance and DHARMa packages (Lüdecke et al. 2021; Hartig 2024) to evaluate residual dispersion, zero-inflation and overall fit. Model selection was based on the Akaike Information Criterion corrected for sample size (AICc; Burnham and Anderson 2002).

Additionally, and to unravel if there was any density dependence in the behavior frequency, we fitted a GLM with a Poisson distribution for each species to analyze the effect of abundance on behavior frequency.

To test if the relative attractiveness of water or polarization cues varied along the polarization gradient, we performed a community-level analysis including the 17 species. We fitted a set of 11 GLMMs (negative binomial, log link), comprising all combinations of the fixed effects of polarization category, water and surface type along with their pairwise interactions using species and site as random intercepts (Table S1 in Material). Individuals were not included as a random effect because odonates were not uniquely marked and thus could not be treated as independent. Model selection was based on AICc, following the approach described above.

### Temperature measurements and analysis

To elucidate if the experimental surfaces reached temperatures that could pose thermal danger to the odonates, the temperatures of the 4 treatment trays along with the temperature of the natural body of water in each site and the temperatures of the soil exposed to the sun and under the shade were taken 15 min after the set-up of the experiment around 11:00 h, at the end of the first 50 min observations and a third time at the end of the second observation with a thermocouple (FLUKE, 51II).

The means of the temperatures measured for each of the 4 treatments along with the soil temperature in the sun and under the shade along with the water temperature in the lotic or lentic water bodies of each site were compared using a Kruskal-Wallis test since the data could not be normalized. The multiple mean comparison was made using the Conover-Iman test.

## Results

In the experimental observations, 11 species of dragonflies and 6 of damselflies performed reproductive behaviors in at least one of the treatment surfaces. The total number of individuals recorded was 136, of which 104 (78%) were dragonflies and 32 (22%) were damselflies. The majority (86%) of the observed individuals belonged to only 4 species: *Orthemis ferruginea, O. discolor*, *Erythrodiplax funerea*, and *Telebasis salva.* None of these species occurred in all 4 sampling sites comprising the polarization gradient. Practically all recorded individuals were males (130), although 6 females of *Erythrodiplax funerea*, *Pantala flavescens*, *Argia tezpi*, *Telebasis filiola*, and *T. salva* were seen perching in at least one of the treatments. A female of *Pantala flavescens* was even seen ovipositing in the 2 treatments with water (Waterpol and Watermetal).

**Figure 3:**
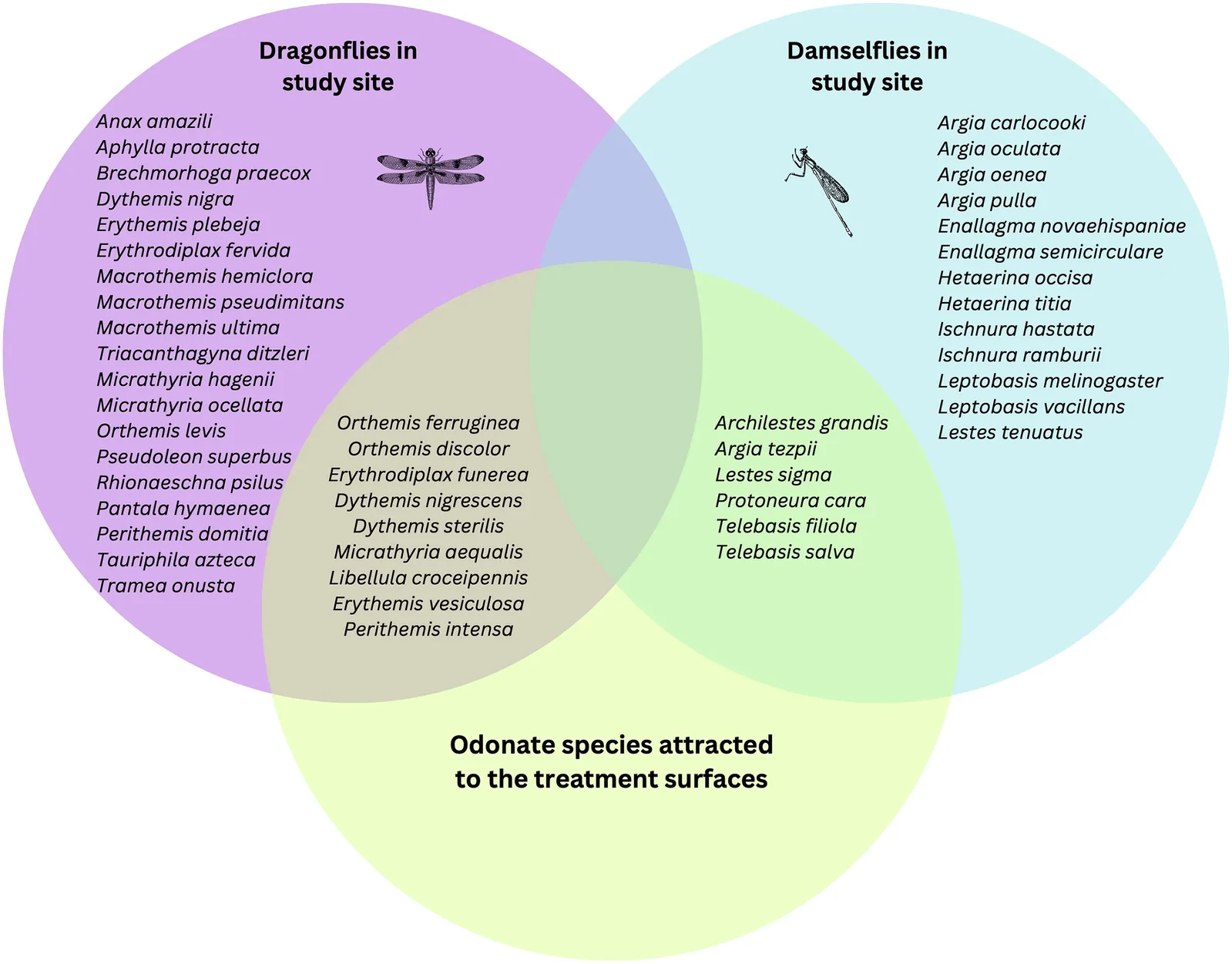
Venn diagram showing the dragonfly and damselfly species registered in the study sites along with the subsets of both suborders attracted to the experimental treatments, all recorded on the greenpol surface.

The species that showed an attraction to the experimental surfaces were always a subset of the total number of species that inhabited each sample site (Fig. 3). Some species of odonates even flew near the experimental trays but were never seen exhibiting behaviors that indicated attraction to the treatment surfaces. The site with null artificial polarization exposure (Chamela Cuixmala reserve) was the site with the highest odonate species richness recorded which contrasted with the highest exposure site where only *Orthemis ferruginea* was recorded visiting the trays.

**Figure 4:**
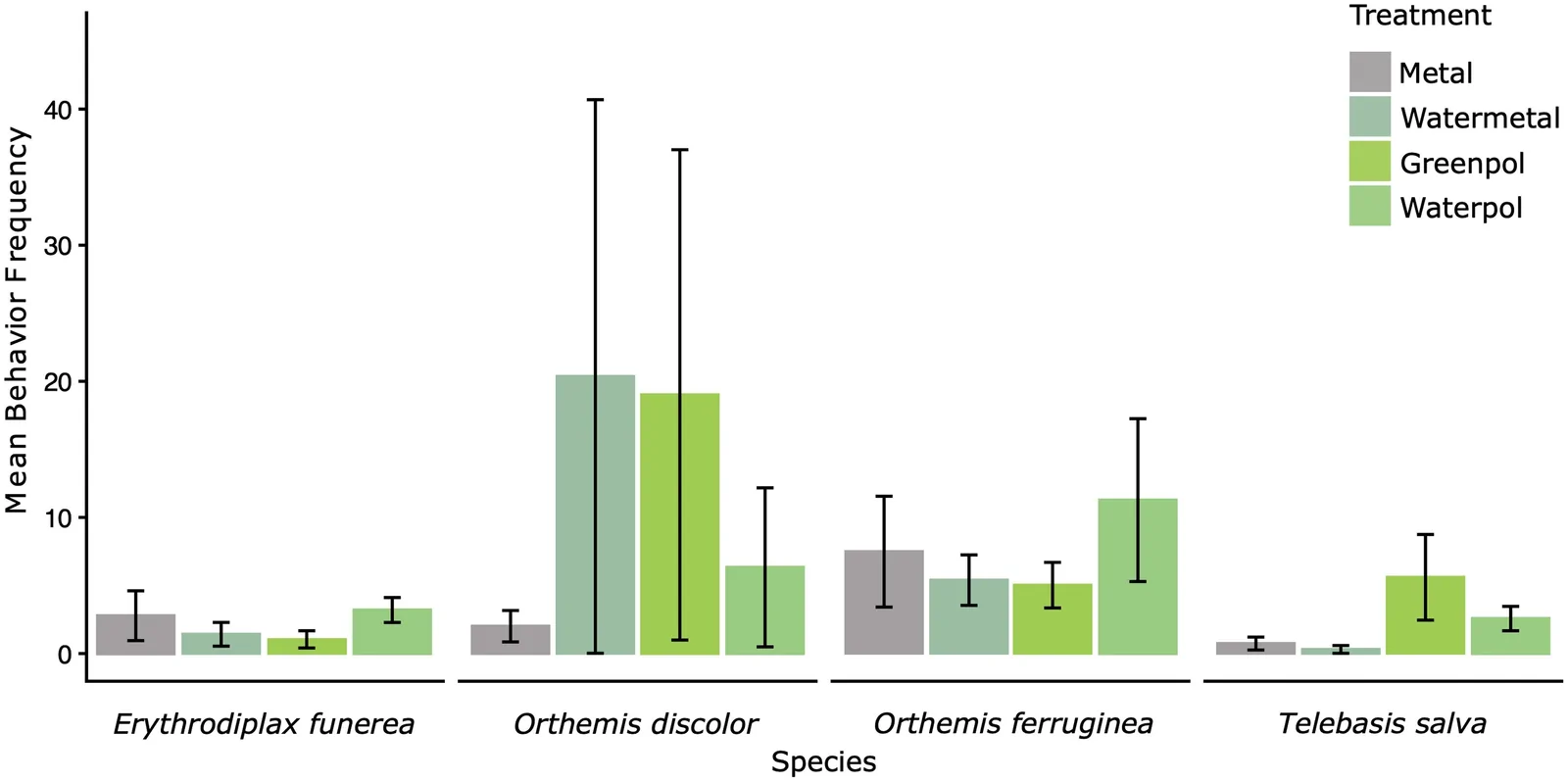
Mean number of behaviors by 4 odonate species: *erythrodiplax funerea*, *orthemis discolor*, *orthemis ferruginea,* and *telebasis salva* performed on 4 experimental test surfaces: waterpol (ie metal tray painted green with water), Greenpol (ie metal tray painted green), Watermetal (ie stainless-steel sheet covered with water) and Metal (ie stainless-steel sheet). Bars represent standard error.

When comparing the frequency of behaviors performed in the experimental surfaces by the 4 most abundant species, we found that both *E. funereal* and *O. discolor* showed lack of preference for any of the treatments (Fig. 4), since the null GLMM showed the best fit with the highest weights (0.86 and 0.69 respectively; Table S2 in Material) for both species subsets. Contrastingly, the model including only polarization category as fixed effect had the best fit for *Orthemis ferruginea* with a weight of 0.36. Models including water or surface type alone had slightly higher AICc values (ΔAICc = 1.1 − 1.4), while the null model performed substantially worse (ΔAICc = 10.5). This suggests that polarization category explains variation in *O. ferruginea’*s behavioral frequency better than surface or water, although a higher behavior count in moderately high polarization exposure showed only marginal significance (*P* = 0.08). Lastly, the surface model showed the best fit for *Telebasis salva* with a weight of 0.38, exhibiting an increase in behavior counts in the green polarizing surface compared with the metal, although this difference was marginally significant (*P* = 0.057). The null model was found to be indistinguishable from the surface model, so preference for the green surface must be interpreted with caution.

Regarding density-dependence, we found a significant negative effect of abundance on the behavior frequency of *O. ferruginea* (estimate = −0.026, S.E. = 0.012, *P* = 0.024), a pattern which was also found for *E. funerea* (estimate = −0.05, S.E. = 0.02, *P* = 0.02; Figure S1 in Material). Meanwhile, *O. discolor* showed the opposite pattern, significantly increasing the frequency of its behaviors in higher abundances (estimate = 0.95, S.E. = 0.29, *P* = 0.001). In the case of the damselfly *T. salva*, no significant density dependence was observed.

Regarding the community analysis, there was no strong evidence that the tested predictors improved model fit relative to the null model. The category only model, along with surface only and the additive model between category and surface were all indistinguishable from the null model (ΔAICc < 2; Table S3 in Material), indicating that the effects of these variables on behavior count are weak or uncertain.

**Figure 5:**
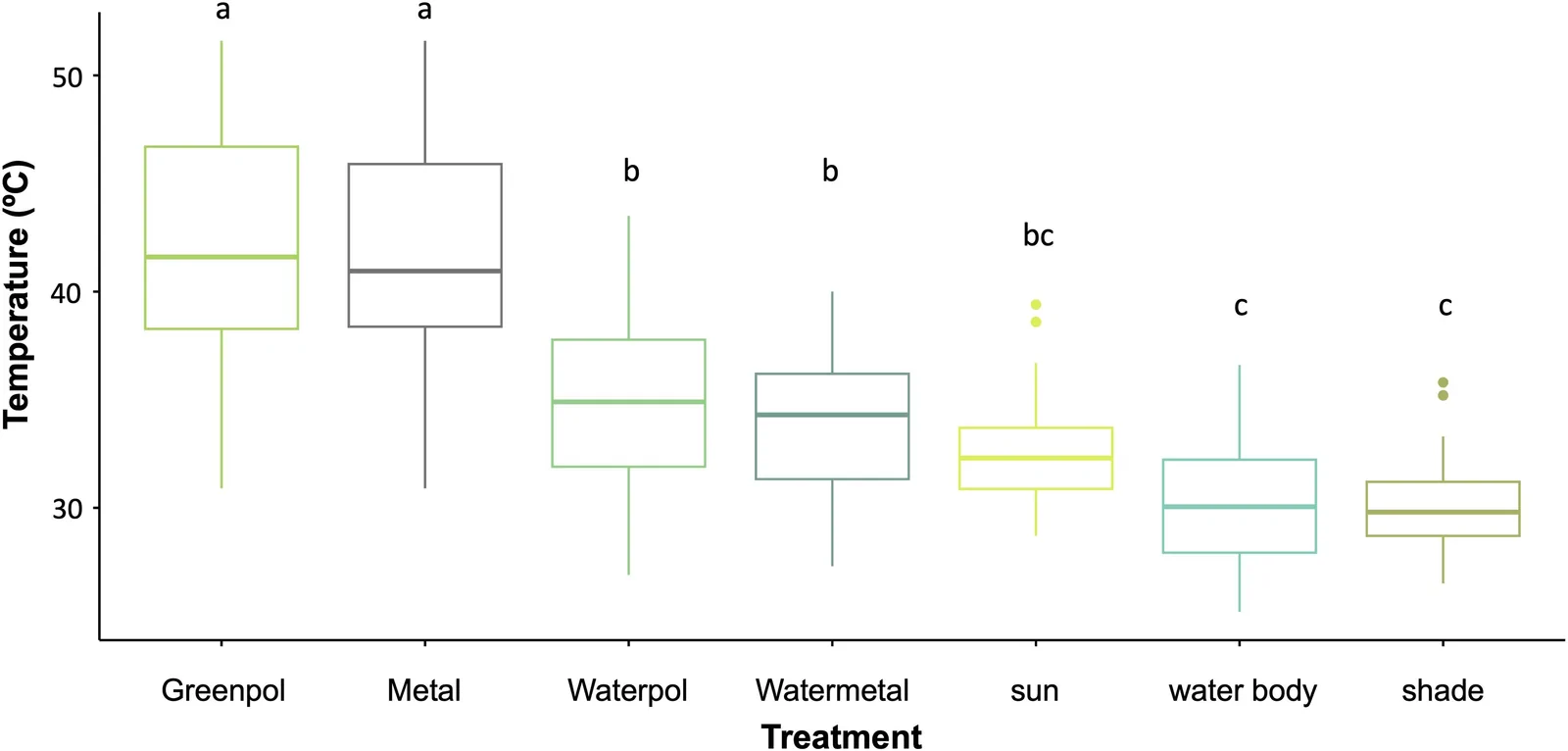
Temperatures of the 4 treatment surfaces: greenpol (ie metal tray painted green), Metal (ie stainless steel sheet), Waterpol (ie metal tray painted green with water), and Watermetal (ie stainless steel sheet covered with water) along with 4 ambient temperatures: sun (ie sun exposed soil temperature), water body (ie water temperature at the sites) and shade (ie shaded soil temperature) taken at each of the study sites. Letters in the middle of 2 box plots indicate a significant difference in the mean temperatures between that pair of surfaces and the others (*P* < 0.05) according to the pairwise Conover-Iman test.

We found important temperature differences between treatments, indicated by the significant differences across treatment temperatures found by the Kruskal-Wallis test (χ^2^ = 270.8, df = 6, *P* < 0.0001). The Greenpol and Metal treatments had the highest mean temperatures, and their mean values did not differ from each other (being 42.2 y 42.1 °C, respectively). However, both differed significantly from the 2 water treatments (Waterpol and Watermetal), whose means were 34.9 and 33.9 °C respectively (Fig. 5). The highest temperature recorded was in the Metal treatment which rose to 51.6 °C and the lowest temperature of 26.8 °C was recorded in a shallow pool. The soil temperatures taken in the sun had a mean of 32.5 °C that differed significantly from those taken under the shade and inside the lotic and lentic water bodies of each site, which were 30.1 and 30.2 °C, respectively.

The Metal tray was the only surface on which dead insects were collected after the experimental observations. Seven female beetles were attracted by the Metal treatment and were found dead, most likely due to heat shock. The species were identified as: *Tropisternus brevicollis* (Hydrophilidae), *Tropisternus calybeus* (Hydrophilidae), *Tropisternus apicipalpis* (Hydrophilidae), and *Laccophilus fasciatus* (Dystiscidae). A water bug belonging to the genus *Pelocoris* sp. (Naucoridae) not identified to the species level was also collected.

## Discussion

In this study, we examined the differences in the attractiveness of polarized light cues compared with other sensory cues associated with water perception in odonates. Our findings revealed significant interspecific variations, suggesting differences in sensory capacities and habitat selection criteria among species. We also observed notable temperature discrepancies between our study conditions and the surrounding environment, leading us to infer potential thermal harm to odonates due to the excessive heating of the ecological traps. Additionally, our investigation indicates some evidence of heightened vulnerability in odonate communities exposed to artificial polarization from urbanization. These results will be discussed in more detail in the following section.

Polarized light associated with human-made materials is a main threat for odonates, which often fall in these traps and defend unviable oviposition habitats. Differences between the relative attractiveness of polarization visual cues and other sensory cues associated with water presence were evaluated for 4 species representing the vast majority of behavioral observations. Of these, *T. salva* was a damselfly while the 3 remaining species (*O. ferruginea*, *O. discolor*, and *E. funerea*) were dragonflies. The incidence of damselflies in ecological traps has been reported less in comparison to dragonflies (Horváth et al. 1998, 2009). Additionally, damselflies are more sensitive to urbanization than dragonflies, and damselfly communities have a reported decrease in abundance and richness under higher urbanization (Suárez-Tovar et al. 2022).

When we analyzed the data for these 4 recurring species, important trends were observed regarding their specific treatment surface preferences. The 3 dragonfly species used all of the treatment surfaces and could be regarded as opportunists concerning their choice of breeding sites. This result coincides with their documented ability to explore new habitats and exploit emerging resources (Corbet 1999). Neither *E. funereal* nor *Orthemis discolor* showed a marked rise in behavior frequency in response to polarization category, surface type or water presence. *Orthemis ferruginea*, the only species recorded in the highest polarization category, showed a marginally significant rise in behavior frequency under moderate polarization exposure, but no changes in behavior frequency regarding the different polarizing surfaces.

Interestingly, the best fit model for *T. salva* showed a marginally significant preference for the Greenpol tray, suggesting that water-related cues are not the most important requisite for choosing a habitat. This damselfly regularly perched directly on the Greenpol tray while the other species mainly used the wooden perches. *T. salva* is a species attracted to vegetation and is regularly found perching on the leaves and stems of aquatic plants and grass, where males exhibit reproductive behaviors (Robinson and Frye 1986; Blinn and Runck 1992). This microhabitat use could explain its choice for the Greenpol tray and why it perched directly on its surface. Our observations suggest that this color appeals to the damselfly, resulting in its attraction to an ecological trap.

Regarding temperature, the Greenpol treatment, being a metal tray painted green, was, along with the Metal treatment, 1 of the 2 hottest surfaces. These temperatures surpassed the thermal limits of all adult odonates studied in this region, spanning from 38 to 43 °C (Castillo-Pérez et al. 2022). Specifically, this region’s critical thermal maxima registered for *O. ferruginea* and *E.* funerea ranged from 39 to 45 °C and 43 to 44 °C, respectively. Due to the high temperatures of these 2 treatments, it is surprising that the damselfly *T. salva* perched directly on the Greenpol tray for minutes at a time, raising the question of what possible effects this exposure has on the physiology of this species. Evidence of dragonflies perceiving high treatment temperatures could be that they perched on the wooden sticks above these surfaces, a behavior that buffers the rise in body temperature that they could experience if they perched directly on the trays.

Three of the 4 odonate species that composed 85% of the individuals who made use of the experimental surfaces had both red and pinkish colorations. The ommochromes in the epidermis of odonates are responsible for conferring red hues (Chapman 1982) and these have been associated with antioxidant properties that could inhibit light damage to the bodies of these insects (Needham 1974). Suárez-Tovar et al. (2023) reported a correlation between red tones in dragonflies and urbanization tolerance, while a rise in the frequencies of red androchromes was reported for a Hawaiian damselfly along a solar radiation exposure gradient (Cooper 2009). Two species, the dragonfly *O. discolor* and the damselfly *T. salva*, were actually categorized as urbanization-tolerant species in a worldwide analysis of urban sensitivity and tolerance that includes 175 species (Suárez-Tovar et al. 2023). This classification resulted from their prevalence in urbanized landscapes compared with more conserved sites. Our results related to O. discolor contrast with this previous analysis since this species was absent at moderate and high exposure sites where *O. ferruginea* persisted. The previously reported urbanization tolerance of *T. salva* explains why this species stood out as the only damselfly species occupying the experimental surfaces in the first 3 of the 4 gradient categories. But precisely because of this, it is a species potentially vulnerable to the loss of individuals exposed to these ecological traps for extended periods.

Contrary to our predictions, the frequency of reproductive behaviors carried out by the individuals of the odonate community in the experimental surfaces, specifically in the ecological trap, was not higher in the conserved site compared with the sites with higher polarization exposure. Following these results, the evidence supporting the existence of a strong selective pressure exerted by anthropogenic polarizing materials and leading to a behavioral adaptation of avoidance of these surfaces is insufficient. One possible explanation for these observations is that selective pressure is weak: very few individuals fall into these traps, and those that do—or that briefly contact the surfaces—can still reproduce during their lifetimes. Under this scenario, the traps do not cause immediate death, and choosing a trap does not necessarily exclude selecting other habitats at different times in an odonate's life. Modeling of the ecological and evolutionary consequences of ecological traps has predicted that even though they reduce population size, traps arising in landscapes where high-quality habitats remain can cause a reduced selection compared with scenarios where high-quality habitats are degraded but still preferred by the organisms; thus, making populations more vulnerable to extinction (Fletcher et al. 2012). This could be the case for the communities at our study sites, and certainly, is in the case of the conserved site. Still, since we didn’t test the water quality at the sites subjected to urbanization, we cannot be certain to what degree these water bodies constitute high-quality habitats.

The resilience of Odonata has been the subject of considerable interest, with numerous studies highlighting this insect order’s capacity to adapt and persist across various environmental gradients. For instance, Suárez-Tovar et al. (2022) reported a surprising finding: while damselfly species richness and abundance exhibited a decrease along an urbanization gradient, there were no discernible differences in dragonfly community diversity along this gradient. Moreover, the study by Suárez-Tovar et al. (2024) revealed that both dragonflies and damselflies exhibited heightened rates of prey capture in highly urbanized areas. These findings highlight the remarkable resilience of odonates, both Zygoptera and Anisoptera, in the face of various environmental pressures and the generalized insect decline worldwide (Goulson 2019).

Another possible explanation for the lack of differences along the gradient we studied is that the selective pressure has already occurred for some of these species. This could have resulted in communities that present species which not only show no attraction to the ecological traps, but also evade their surfaces. Natural selection could have already favored species that effectively avoid erroneous habitats, as seen in some moth species where a reduced flight-to-light response mitigates population declines as opposed to species with strong polarotaxis (Altermatt and Ebert 2016; van Langevelde et al. 2018).

A third possibility is that attraction to polarization is a strongly canalized trait for odonates, but additional criteria come into play when odonates choose their habitat. The observed pattern of avoidance of the polarizing trap may stem from odonate prioritization of alternative habitat cues and stricter choice criteria: factors such as preference for greater water depth, larger water body size, lower water temperature, among others, may take precedence over attraction to polarized light. The antennae of odonates, while small, have been found to have thermo- and hygro-sensitive capacities (Piersanti et al. 2011) along with olfactory neurons (Rebora et al. 2012), so water quality assessed by smell could be playing an important role as in the damselfly *Ischnura elegans* which has been found to prefer conspecific nymph rearing or plankton-containing water over tap water as an oviposition site (Frati et al. 2016). Consequently, odonates exhibiting such preferences may not be captured by the traps, accounting for their absence from our observations. Along with this same reasoning, the fact that almost no female odonates were observed on the treatment surfaces is noteworthy and strongly suggests that males are more prone to being driven by ecological traps. This could be attributed to females having more strict criteria for the selection of oviposition sites, given their higher fitness costs compared with males.

Finally, the frequency of behaviors in the ecological traps can be understood in terms of density dependence. In this regard, *O. ferruginea* and *E. funerea* decreased the number of behaviors in the experimental trays when individual density was higher. Although they are territorial species, they show a decrease in competition when their numbers are high. Conversely, we observed that *O. discolor* increased the number of behaviors in higher-density situations indicating that territoriality is a density-dependent trait that increases competition for the most optimal habitats. Meanwhile, *T. salva* did not show a density-dependent trend and did not strongly defend the selected habitat, which could be because it is not categorized as a territorial species (IUCN 2000). Moreover, 2 or more males were often seen perching simultaneously on the same tray without confrontations (VSG, personal observations). These results show that individual density can be a factor that influences trap visitation frequency in odonates. Species like *O. discolor* that show higher behavior frequencies at higher densities could decrease individual exposure to the traps since it is more difficult for a single odonate to remain in the trap for long periods of time. However, these individuals would lose energy reserves while actively fighting for a suboptimal habitat. Meanwhile, species that do not exhibit a rise in competitiveness at higher densities favor increased individual exposure to the adverse effects of the ecological traps. Kokko and Sutherland (2001) report a behaviorally mediated Allee effect produced by ecological traps where population growth is reduced at lower species densities since more individuals are able to choose their favored habitat under reduced competition. This could produce local extinctions where individual density is already low, such as that registered in our more conserved sites.

Anticipating which species are more vulnerable under different conditions can provide valuable insights into the threats faced by insect populations and their viability in human-altered environments (Wagner et al. 2021). While further research is needed to fully understand the costs of defending ecological traps at different individual odonate densities, existing evidence strongly suggests the need for caution and proactive measures to mitigate harm to aquatic habitats. Our findings emphasize the importance of addressing light pollution in the vicinity of water bodies as a critical strategy for protecting and conserving odonates and other aquatic insects in the face of ongoing environmental change. By taking decisive action to reduce light pollution, we can play a vital role in safeguarding the essential components of aquatic ecosystems for future generations.

## Supplementary Material

arag108_Supplementary_Data

## Data Availability

Analyses reported in this article can be reproduced using the data provided by authors (Sandoval Granillo and Córdoba-Aguilar 2026).
