# Supplementary material for: Attraction to water and polarization in dragonflies and damselflies along a light pollution gradient
Source: Behav Ecol. 2026 Aug 27;37(6):arag108. doi: 10.1093/beheco/arag108 (PMC13595255; doi:10.1093/beheco/arag108)
Supplement: Supplementary file 1 [file arag108_supplementary_data.docx]

SUPPLEMENTARY MATERIAL

**Table S1**. List of 11 GLMMs fitted for reproductive behavior count data (*B*) of the odonate community in response to combinations of the following fixed variables: polarization gradient category (*P*), water presence (*W*) and treatment surface type (*S*). *υ*_0_ denotes the random intercept of site and γ_0_ refers to the random intercept of individual species.

| Model | Notation |
| --- | --- |
| 1 (full additive) | *B* = β_0_ +  β_1_*P* +  β_2_*W +* β_3_*S+ υ*_0_+ γ_0_ |
| 2 (polarization and water interaction) | *B* = β_0_ +  β_1_*P* +  β_2_*W +* β_3_(P× *W)+ υ*_0_+ γ_0_ |
| 3 (polarization and surface interaction) | *B* = β_0_ +  β_1_*P* +  β_2_S *+* β_3_(P× *S)+ υ*_0_+ γ_0_ |
| 4 (water and surface interaction) | *B* = β_0_ +  β_1_*W* +  β_2_*S +* β_3_(W× *S)+ υ*_0_+ γ_0_ |
| 5 (polarization and water additive) | *B* = β_0_ +  β_1_*P* +  β_2_*W + υ*_0_+ γ_0_ |
| 6 (polarization and surface additive) | *B* = β_0_ +  β_1_*P* +  β_2_*S + υ*_0_+ γ_0_ |
| 7 (water and surface additive) | *B* = β_0_ +  β_1_*W* +  β_2_*S + υ*_0_+ γ_0_ |
| 8 (polarization only) | *B* = β_0_ +  β_1_*P* *+ υ*_0_+ γ_0_ |
| 9 (water only) | *B* = β_0_ +  β_1_W *+ υ*_0_+ γ_0_ |
| 10 (surface only) | *B* = β_0_ +  β_1_S *+ υ*_0_+ γ_0_ |
| 11 (null) | *B* = β_0_ *+ υ*_0_+ γ_0_ |

**Table S2**. Diagnostics for the GLMMs fitted for the four most abundant species according to the Akaike Information Criterion corrected for sample size (AICc), along with ΔAICc (difference between model AICc and the AICc of the best-fit model) and model weights.

| Model | AICc | ΔAICc | Weight |
| --- | --- | --- | --- |
| *Erythrodiplax funerea* | | | |
| Interaction (water and surface) | 91.0 | 10.93 | 0.004 |
| Additive (water and surface) | 89.6 | 9.55 | 0.007 |
| Polarization category only | 85.0 | 4.97 | 0.07 |
| Surface only | 86.0 | 5.94 | 0.043 |
| Water only | 86.0 | 5.94 | 0.043 |
| Null | 80.1 | 0.00 | 0.834 |
| *Orthemis discolor* | | | |
| Interaction (water and surface) | 87.2 | 15.07 | 0.000 |
| Additive (water and surface) | 82.9 | 10.8 | 0.003 |
| Polarization category only | 76.1 | 4.05 | 0.091 |
| Surface only | 76.7 | 4.60 | 0.069 |
| Water only | 76.6 | 4.55 | 0.071 |
| Null | 72.1 | 0.00 | 0.693 |
| *Orthemis ferruginea* | | | |
| Interaction (water and surface) | 241.4 | 6.20 | 0.016 |
| Additive (water and surface) | 238.8 | 3.69 | 0.057 |
| Polarization category only | 235.2 | 0.00 | 0.359 |
| Surface only | 236.3 | 1.14 | 0.203 |
| Water only | 236.5 | 1.36 | 0.182 |
| Null | 245.7 | 10.53 | 0.002 |
| *Telebasis salva* | | | |
| Interaction (water and surface) | 110.2 | 6.76 | 0.013 |
| Additive (water and surface) | 106.6 | 3.22 | 0.076 |
| Polarization category only | 109.0 | 5.58 | 0.023 |
| Surface only | 103.4 | 0.00 | 0.382 |
| Water only | 106.5 | 3.10 | 0.081 |
| Null | 103.6 | 0.21 | 0.343 |

**Table S3**. Diagnostics for the GLMMs fitted for the odonate community according to the Akaike Information Criterion corrected for sample size (AICc), along with ΔAICc (difference between model AICc and the AICc of the best-fit model) and model weights.

| Model | AICc | ΔAICc | Weight |
| --- | --- | --- | --- |
| 1 (full additive) | 651.4 | 3.29 | 0.047 |
| 2 (polarization and water interaction) | 656.1 | 8.00 | 0.004 |
| 3 (polarization and surface interaction) | 655.5 | 7.41 | 0.006 |
| 4 (water and surface interaction) | 653.0 | 4.98 | 0.020 |
| 5 (polarization and water additive) | 650.3 | 2.20 | 0.081 |
| 6 (polarization and surface additive) | 649.1 | 1.08 | 0.142 |
| 7 (water and surface additive) | 651.5 | 3.41 | 0.044 |
| 8 (polarization only) | 648.3 | 0.27 | 0.212 |
| 9 (water only) | 650.4 | 2.31 | 0.077 |
| 10 (surface only) | 649.4 | 1.37 | 0.123 |
| 11 (null) | 648.3 | 0.00 | 0.244 |

**Fig S1.** Effect plots of the GLMs with abundances as the predictor variable and behavior frequency as the response variable for each of the four most abundant species. Asterisks indicate a significance of *p*< 0.05 in the effect of abundance on behavior frequency.
